# Quantitative impacts of climate change and human activities on grassland growth in Xinjiang, China

**DOI:** 10.3389/fpls.2024.1497248

**Published:** 2025-01-22

**Authors:** Hanyi Rui, Beier Luo, Ying Wang, Lin Zhu, Qinyuan Zhu

**Affiliations:** ^1^ Nanjing Institute of Environmental Sciences, Ministry of Ecology and Environment, Nanjing, China; ^2^ College of Geography and Remote Sensing, Hohai University, Nanjing, China; ^3^ School of Tourism and Social Management, Nanjing Xiaozhuang University, Nanjing, China

**Keywords:** grassland, climate change, human activities, Xinjiang, NDVI

## Abstract

Grassland is an important vegetation type in Xinjiang, China, playing a crucial role in the terrestrial carbon cycle. Previous studies have shown that both climate change and human activities significantly impact grassland growth. However, research quantifying the contributions of these two factors to grassland changes is still not thorough enough. This study utilized remote sensing data, i.e., Normalized Difference Vegetation Index (NDVI), to analyze the spatial trends of grassland changes from 1982 to 2015, and the correlation between NDVI and climate factors. Then, relative contributions of climate change and human activities to grassland changes were explored across Xinjiang. The results indicated that there was a significant spatial heterogeneity in the interannual variations of NDVI in the study area, showing an overall increasing trend (covering 62.5% of the study area). This was mainly attributed to the warming and humidifying trend of Xinjiang’s climate in recent decades, where increased precipitation and rising temperatures promoted grassland growth. The main regions with increased NDVI included the western part of Changji Hui Autonomous Prefecture, the southern part of Tacheng Prefecture, and the northwestern part of the Tarim Basin; while the areas with decreased NDVI were mainly located in the western part of the study area, e.g., the Ili River basin, and the Tekes River basin. Compared to precipitation, NDVI showed a stronger correlation with temperature, which was related to temperature promoting organic matter decomposition and enhancing vegetation nutrient utilization efficiency. NDVI was negatively correlated with VPD, mainly due to the effects of transpiration and surface evaporation. In terms of grassland growth, climate change (52%) contributed as much as human activity (48%). For the grassland reduction, human activities played a larger role. Overall, in mountainous and flat areas, human activities contributed more (64.29%) than climate change (35.71%), including activities such as grazing and urbanization.

## 1 Introduction

Grassland is an important component of the terrestrial ecosystem in our country, with functions such as sand fixation, dust prevention, water conservation, climate regulation, and playing a key role in biodiversity conservation and carbon sink function maintenance (Xu et al., 2022). Climate change and human activities are the main driving factors for changes in terrestrial ecosystems (Zhang et al., 2024), which have always been the focus of fields such as ecology (Zheng et al., 2019). Climate change includes temperature, precipitation, and vapor pressure deficit (VPD) (Yuan et al., 2019). Among them, temperature and precipitation are the dominant factors affecting processes such as seed germination and seedling growth, and have important impacts on vegetation distribution and carbon balance (Xu et al., 2019, 2016). VPD is defined as the difference between saturated water vapor pressure and actual water vapor pressure at a specific temperature, and is an important driving factor for plant demand for atmospheric water (Rawson et al., 1977). The impact of human activities on grasslands has also been widely studied (Hilker et al., 2013; Li et al., 2017). For example, the “settled pastoralists project” and the “national grazing ban and grassland restoration project” have improved the grassland areas in Xinjiang, China, but overgrazing has led to grassland degradation in the Ili River Valley and Tacheng area (Yang et al., 2014). Accurately assessing the impact of climate change and human activities on grassland ecosystems will help the government formulate ecological protection policies and provide theoretical basis for grassland management, utilization, and protection.

Normalized Difference Vegetation Index (NDVI) has been widely used in vegetation remote sensing (Defries and Townshend, 1994; Detsch et al., 2016; Huang et al., 2010), gradually maturing in the analysis of vegetation growth, yield estimation, and land desertification research, becoming an important tool for analyzing changes in land surface cover and their response to climate change and human activities (Li and He, 2009; Jiang et al., 2021; Li et al., 2022). NDVI is sensitive to global climate change, especially in arid and semi-arid areas where it exhibits high sensitivity to precipitation (Eklundh, 1998; Piao et al., 2004, 2003). Human activities also have a strong impact on NDVI (Hilker et al., 2013).

Currently, many scholars have conducted extensive research on the factors of climate change and human activities inducing NDVI changes using remote sensing methods, but further research is needed to quantify the contributions of climate change and human activities to vegetation changes (Sun et al., 2015; Wang et al., 2015; Xie et al., 2016). Residual analysis is a common quantitative analysis method that quantifies the contribution of climate change by establishing an equation between NDVI and climate factors (Li et al., 2017; Wang et al., 2015). This method has achieved good results in distinguishing and quantifying the climatic and anthropogenic impacts on vegetation dynamics in the central and northern regions of China and the Yulin region in northwest China (Sun et al., 2015; Jiang et al., 2017).

Xinjiang Uygur Autonomous Region is a hotspot for global climate change research. Grasslands, as an important vegetation type, play a crucial role in maintaining ecological balance and ensuring the region’s climate and economic health. Considering the impact of climate change and human activities on grasslands, this study takes Xinjiang’s grasslands as the research object, using the GIMMS NDVI3g dataset, GLASS-GLC dataset, and Vegetation Continuous Fields (VCF) time series data to analyze the changing trends of Xinjiang’s grasslands, elucidate the driving factors of grasslands and their correlations, and explore the quantitative contributions of climate change and human activities to grasslands. This will contribute to the rational utilization of grassland resources in Xinjiang and the protection of the ecological environment, while providing a scientific basis for understanding vegetation evolution and predicting the changing characteristics of vegetation under climate and human influences.

## 2 Materials

### 2.1 Study area

Xinjiang Uygur Autonomous Region is located between 73°40′ to 96°18′ east longitude and 34°25′ to 48°10′ north latitude. It is the largest provincial-level administrative region in China with a total area of approximately 1.6649 million square kilometers, accounting for one-sixth of China’s land area. It is a region with diverse and harsh environments (Zhang and Zhang, 2023). The Altai Mountains are in the north, while the Kunlun Mountains, Altun Mountains, and Tianshan Mountains are in the south. The Tianshan Mountains run through the central part, forming the Tarim Basin in the south and the Junggar Basin in the north.

Xinjiang is located deep inland, surrounded by high mountains on all sides, far from the ocean, which limits the arrival of maritime air currents and forms a distinct temperate continental climate. Under this climate condition, Xinjiang experiences large temperature variations, significant day-night temperature differences, abundant sunshine, low precipitation, and a dry climate. In Xinjiang, grasslands serve as an important vegetation type, playing a crucial role in ecosystem services such as soil and water conservation, and climate regulation. However, occurrences of drought and overgrazing have negative impacts on Xinjiang’s ecosystems. Therefore, analyzing the changes in Xinjiang’s grasslands and their driving factors is of great significance for studying global and regional changes.

**Figure 1:**
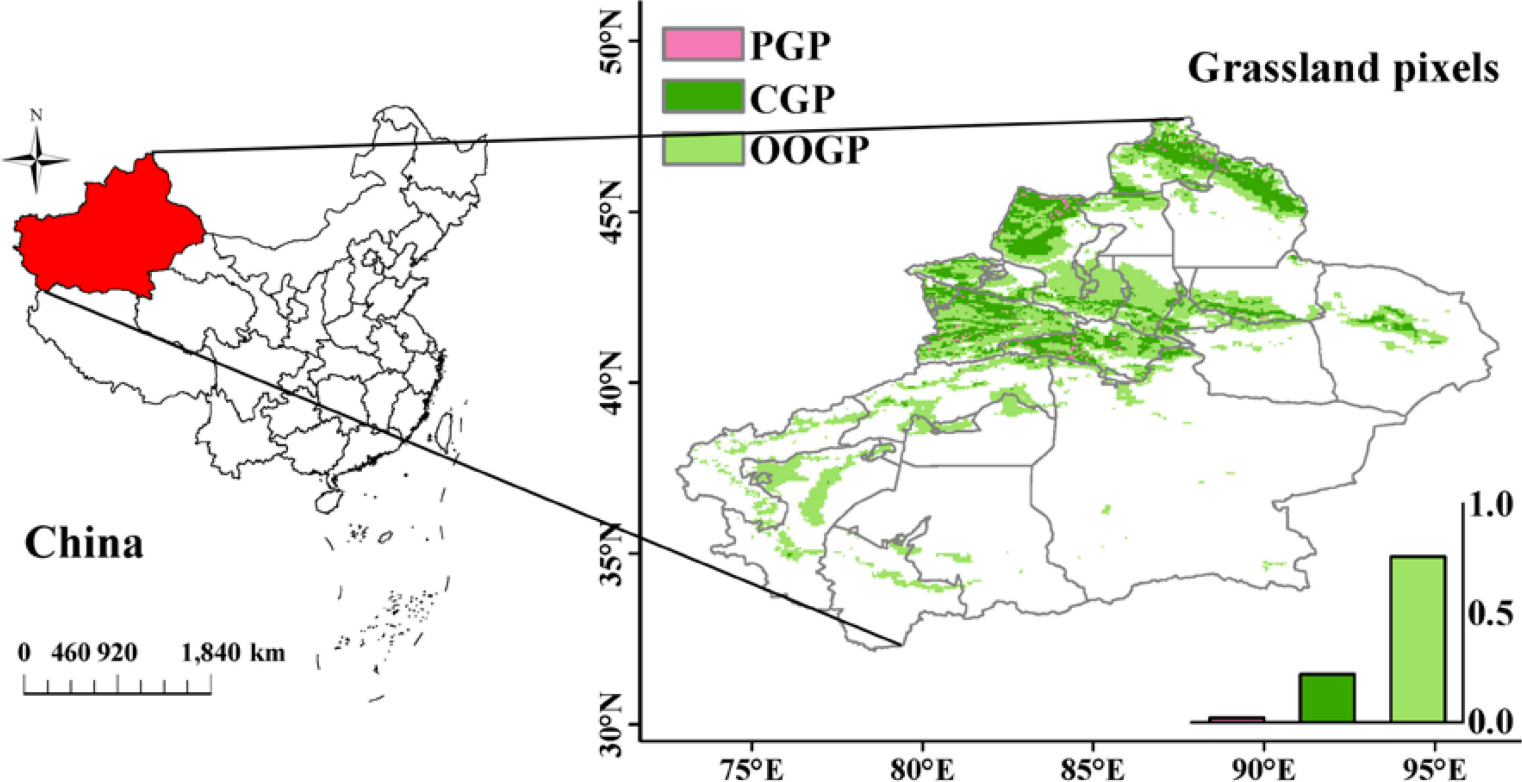
Grassland pixel over the Xinjiang from 1981 to 2015.The colors represent three types of grassland pixels. Light green pixels represent pixels where grassland has appeared at least once from 1981 to 2015 (OOGP), pink pixels represent approximated pure grassland pixels (PGP), and dark green pixels represent pixels that have always been grassland (CGP). The frequency histogram displaying the areal proportions (%) of corresponding regions is inset.

The study area consists of pixels of grassland that appeared at least once from 1981 to 2015 (OOGP) (referred to below), mainly distributed in regions above 40° north latitude, located near the Tianshan Mountains and Altai Mountains, on the edge of the Gurbantünggüt Desert, and the western edge of the Taklimakan Desert (**Figure 1**). The study area covers the main distribution areas of grasslands in Xinjiang, reflecting the interannual variation trends of grasslands in Xinjiang.

### 2.2 GIMMS 3g NDVI dataset

The vegetation index is an indicator that reflects the relative abundance and activity of green vegetation through quantified radiation values. It is commonly used to describe the physiological status, green biomass, and productivity of vegetation in a study area, and is highly sensitive to vegetation growth (Tucker et al., 1986). The GIMMS NDVI3g.V1 dataset (https://ecocast.arc.nasa.gov/data/pub/gimms/3g.v1/) is a long time series of NDVI data obtained by the Advanced Very High Resolution Radiometer (AVHRR) sensor from NOAA. This dataset is global in scope, covering the time range from 1981 to 2015, with a spatial resolution of 1/12° and a temporal resolution of 15 days. This is the NDVI data product with the longest time span currently, widely used in long-term vegetation change studies in different regions. Compared to early AVHRR products, GIMMS NDVI3g has higher accuracy and is less affected by factors such as atmospheric water vapor and volcanic eruptions, hence it has been widely applied in research. Previous studies suggested that the GIMMS NDVI data performed well in detections of grass changes across Xinjiang, China (Liu et al., 2018; Du et al., 2015; Yao et al., 2018).

### 2.3 GLASS-GLC dataset

The GLASS-GLC dataset (https://doi.pangaea.de/10.1594/PANGAEA.913496) records the annual dynamic changes in global land cover from 1982 to 2015 for the first time at a resolution of 5 kilometers. This dataset is generated using the latest version of GLASS CDRs and the Google Earth Engine (GEE) platform. Compared to earlier global land cover products, GLASS-GLC has higher consistency, more details, longer temporal coverage, and more detailed categories. It includes seven categories such as cropland, forest, grassland, shrubland, tundra, barren land, ice, and snow, with an average overall accuracy of 82.81% over 34 years. GLASS-GLC is suitable for long-term analysis of land cover changes, application in Earth system modeling, and promoting research on vegetation dynamics (Liu et al., 2020).

Based on GLASS-GLC data, this article obtained the annual land cover types in Xinjiang from 1982 to 2015. By comparing the land cover types of each pixel over 34 years, it identified pixels where the land cover type has always been grassland (CGP) and pixels where grassland has appeared at least once in the 34 years (OOGP).

### 2.4 Climatic data

The climate data used in this study includes near-surface air temperature (°C), surface precipitation rate (mm h^−1^), near-surface air pressure (Pa), and near-surface specific humidity. These data are sourced from the China Meteorological Forcing Dataset (CMFD, https://poles.tpdc.ac.cn/zh-hans/data/8028b944-daaa-4511-8769-965612652c49/) (1979-2018). The CMFD is produced by integrating conventional meteorological observation data from the China Meteorological Administration with existing international Princeton reanalysis data, GLDAS data, GEWEX-SRB radiation data, and TRMM precipitation data as background fields. The data is in NETCDF format, with a temporal resolution of 3 hours and a horizontal spatial resolution of 0.1°. Compared to internationally published reanalysis data, CMFD has higher spatiotemporal resolution, more comprehensive integration of ground observation data, continuous time coverage, and stable quality. Therefore, it exhibits superior performance in regional climate research and is one of the most widely used climate datasets in China (He et al., 2020).

The formula for calculating VPD (vapor pressure deficit) based on climate data is as follows:


(1) $$q=0.622\frac{e}{P}$$

(2) $$P_{S}=610.78exp\left[\frac{17.269T}{237.3+T}\right]$$

(3) $$VPD=P_{S}-e$$

In the formula, q represents specific humidity, p represents air pressure, e represents actual water vapor pressure, ${\text{P}}_{\text{S}}$ represents saturated water vapor pressure, T represents temperature, and VPD represents atmospheric vapor pressure deficit (Singh et al., 2002).

## 3 Methods

### 3.1 Statistical analysis

Maximum Value Composition (MVC) is commonly used for synthesizing satellite images with revisit cycles to reduce or eliminate the impact of clouds, weather, or other factors on the data (Huete et al., 2002). By processing NDVI data using the MVC method, NDVI raster images at the annual scale of Xinjiang from 1981 to 2015 can be obtained. At the pixel scale, using a simple linear regression method, the annual variation rate of NDVI from 1981 to 2015 can be calculated to depict its temporal trend and change intensity (Gao et al., 2022). This method comprehensively considers the NDVI data of each year within the study period, and the calculation formula is as follows:


(4) $$\theta_{slope}=\frac{n\times \sum_{i=1}^{n}\left(i\times NDVI_{i}\right)-\left(\sum_{i=1}^{n}i\right)\times \left(\sum_{i=1}^{n}NDVI_{i}\right)}{n\times \sum_{i=1}^{n}i^{2}-{\left(\sum_{i=1}^{n}i\right)}^{2}}$$

In the formula, n represents the length of the study period (NDVI data in this study is from 1981 to 2015, n=35); i is the serial number of the study year, with 1981 corresponding to i=1, and so on, up to 2015 corresponding to i=35; ${\text{NDVI}}_{\text{i}}$ represents the NDVI value in year i of the study period; ${\text{θ}}_{\text{slope}}$ is the interannual change rate of NDVI. If ${\text{θ}}_{\text{slope}}$ > 0, NDVI shows an increasing trend, if ${\text{θ}}_{\text{slope}}$ < 0, NDVI shows a decreasing trend.

In order to quantify the relationship between NDVI and temperature, precipitation, and VPD, this study uses Pearson correlation analysis to calculate the single correlation coefficients of NDVI with temperature, precipitation, and VPD at the pixel scale. The calculation formula is as follows:


(5) $$R_{xy}=\frac{\sum_{i=1}^{n}\left[\left(x_{i}-\bar{x}\right)\left(y_{i}-\bar{y}\right)\right]}{\sqrt{\sum_{i=1}^{n}{\left(x_{i}-\bar{x}\right)}^{2}\sum_{i=1}^{n}{\left(y_{i}-\bar{y}\right)}^{2}}}$$

In the formula, ${\text{R}}_{\text{xy}}$ represents the correlation coefficient; ${\text{x}}_{\text{i}}$ is the NDVI for the i-th year; ${\text{y}}_{\text{i}}$ is the temperature, precipitation, and VPD for the i-th year; $\bar{x}$ is the mean value of NDVI during the study period; $\bar{y}$ is the mean value of temperature, precipitation, and VPD during the study period. When $\left|{\text{R}}_{\text{xy}}\right|$ >0.8, it is highly correlated; 0.5< $\left|{\text{R}}_{\text{xy}}\right|$ <0.8 is moderately correlated; 0.3< $\left|{\text{R}}_{\text{xy}}\right|$ <0.5 is weakly correlated; generally, $\left|{\text{R}}_{\text{xy}}\right|$ <0.3 is uncorrelated (Chen et al., 2023).

The partial correlation coefficients between NDVI and concurrent temperature, precipitation, VPD, assuming two variables remain constant, the relationship between NDVI and the other variable is calculated as follows:


(6) $$R_{xy\times z}=\frac{R_{xy}-R_{xz}R_{yz}}{\sqrt{\left(1-R_{xz}^{2}\right)\left(1-R_{xz}^{2}\right)}}$$

In the formula, ${\text{R}}_{\text{xy}\times \text{z}}$ represents the partial correlation coefficient between x and y under the assumption that variable z remains unchanged, and so on.

In order to analyze the fluctuation pattern of vegetation coverage, this study adopted the coefficient of variation method (Milich and Weiss, 2000). The calculation formula is as follows:


(7) $$CV_{NDVI}=\frac{\sigma_{NDVI}}{\bar{NDVI}}$$

In the formula, ${\text{CV}}_{\text{NDVI}}$ refers to the coefficient of variation of NDVI values at a certain time sequence. σ represents standard deviation, and $\bar{NDVI}$ represents the mean, used to evaluate the stability of NDVI in the time series. A larger ${\text{CV}}_{\text{NDVI}}$ value indicates a more dispersed data distribution, greater fluctuation, and unstable time series; conversely, a smaller value indicates a more concentrated data distribution and a relatively stable time series.

Using the F test to determine the significance of interannual variation rate, the formula is as follows:


(8) $$F=K\times \frac{\left(n-2\right)}{M}$$

Among them, K and M are the regression sum of squares and residual sum of squares respectively, with n being the total number of years, which is 35.

### 3.2 Vegetated area of interest

This study aims to establish a purely climate-induced grassland NDVI model using approximated pure grassland pixels (Zhou et al., 2022). For each pixel, this study ignores areas with sparse or non-existent grassland vegetation (areas with annual average NDVI less than 0.1) (de Jong et al., 2011; Weiss et al., 2010). The model is then applied to the entire study area, specifically to pixels where grassland has appeared at least once (OOGP). Finally, the NDVIc derived from the model (the NDVI values influenced by climate change) is subtracted from the observed NDVI data (NDVIo) to obtain the NDVI values influenced by human activities (NDVIh), studying the impact of Climate Change (Cc) and Human Activities (Ha) on grassland vegetation in the study area. The detailed methodology framework of this study is shown in the figure below.

**Figure 2:**
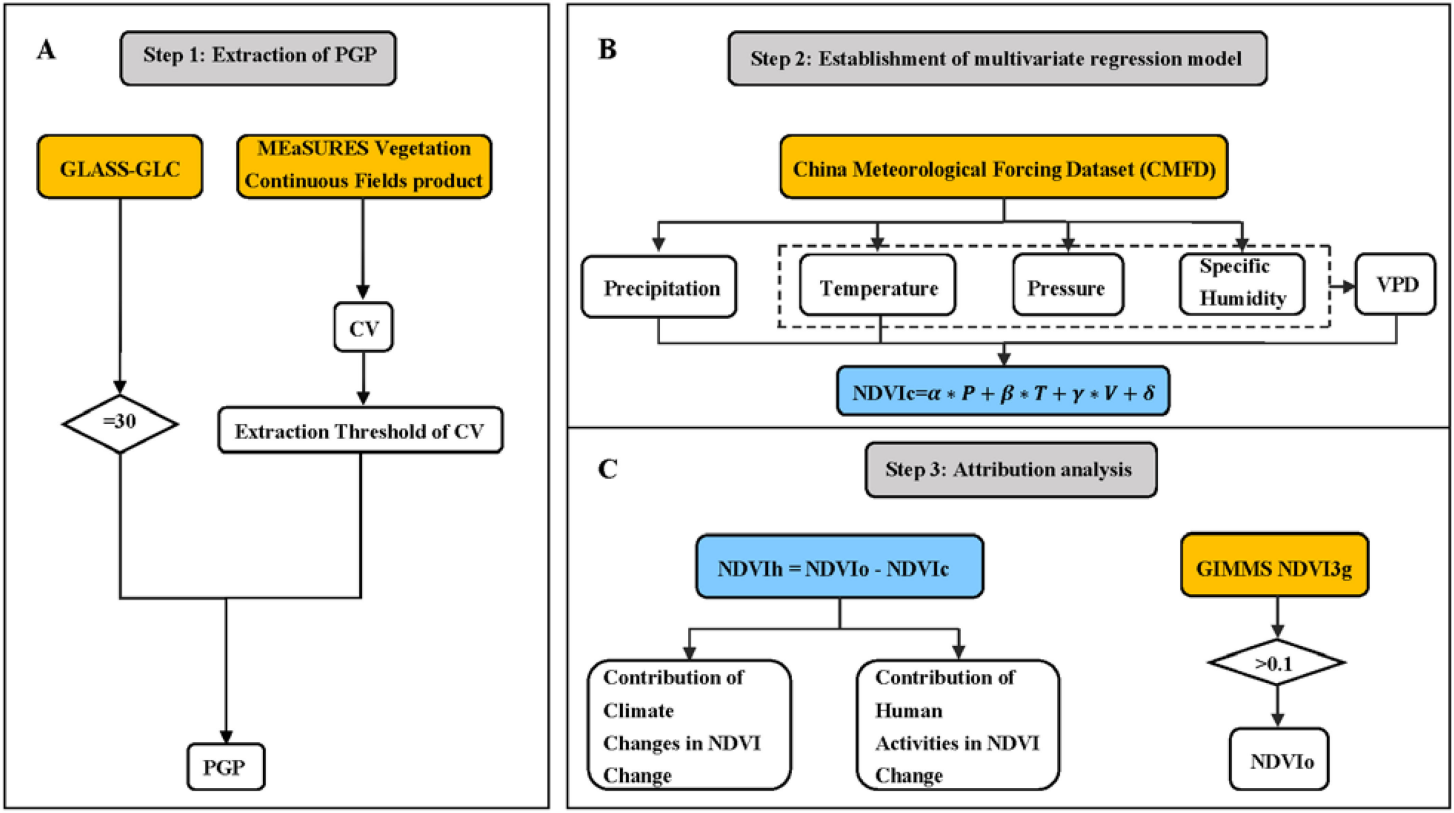
Step 1: Extraction of approximated pure grassland pixels (PGP) **(A)**. Initially, grassland pixels with a value of 30 were extracted from the GLASS-GLC dataset to obtain pixels that were consistently grassland from 1981 to 2015 (Constant Grassland Pixels, CGP). Then, the CV (Coefficient of Variation) values of the CGP were calculated using VCF data, and approximated PGP were identified based on a specified threshold. Step 2: NDVIc was calculated using a multiple regression equation based on precipitation, temperature, and VPD **(B)**. Step 3: In the GIMMS NDVI3g dataset, data greater than 0.1 were considered as observed NDVI (NDVIo). NDVIh represents the difference between NDVIo and NDVIc **(C)**.

Step 1: Extract approximated pure grassland pixels (PGP) (**Figure 2**). Based on the GLASS-GLC dataset and vegetation continuous field (VCF) time series data, a method for extracting approximated PGP was developed by combining grassland pixels (CGP) and coefficient of variation (CV) from 1982 to 2015. CV is the ratio of the standard deviation to the mean of the VCF time series at each pixel, widely used as an indicator to measure the degree of fluctuation, distinguishing grassland pixels affected by climate from those not affected by climate. Therefore, it can be assumed that pure grassland pixels are only influenced by climate change.

Step 2: Establish a multiple regression model (**Figure 2**). Based on the selected PGP, import four climate variables from CMFD: precipitation, near-surface air temperature, surface air pressure, and near-surface air specific humidity. VPD is calculated from temperature, pressure, and specific humidity (Singh et al., 2002). Establish a multiple regression model between NDVI and climate variables (precipitation, temperature, and VPD). Based on this, the NDVIc of each pixel was simulated. Where NDVIc represents climatic grassland; P, T, V represent annual average precipitation, temperature, and VPD, with units in mm, °C, and hPa respectively; α, β, γ represent the regression coefficients of multiple linear regression; δ is the error term.

Step 3: Calculate the impact of human activities on grasslands (**Figure 2**). Based on GIMMS NDVI3g data, obtain observed NDVI (NDVIo). The difference between NDVIc and NDVIo is the residual (NDVIh), indicating the response of grasslands to human activities. Quantitatively analyze the impact of climate change and human activities on grasslands in Xinjiang.

According to the above method, over the past 35 years, the grassland pixel (CGP) has mainly been distributed above 43°N (**Figure 1**). Because the smaller the CV value, the more stable it is. Among the pixels that have always been grassland, the CV values range from 0.6% to 28%. Sorting the CV values from small to large, the 5th percentile is chosen as the threshold (Zhou et al., 2022). Therefore, approximated PGP with CV values less than 4% are selected for modeling (**Figure 1**). Compared to the model based on CGP, the NDVIc obtained from the multiple linear regression model based on PGP (i.e., NDVI induced by Cc) can better reflect the impact of climate on grassland NDVI.

According to the indicators provided in the literature, the correlation coefficient (CC) of the model is 0.496, the bias (BIAS) is -1.2%, and the root mean square error (RMSE) is 0.185 (Mentaschi et al., 2013). Therefore, it can be considered that the model is reasonable and reliable in the study area, and can be used to distinguish the impact of human activities (Ha) and climate change (Cc) on NDVI during the study period. Further analysis revealed that if modeling is done on the same modeling pixel without considering VPD, the CC is 0.475, BIAS is -0.836%, and RMSE is 0.132. If modeling is done using pixels where grassland has appeared at least once in 35 years and considering VPD factors, the CC is 0.6, BIAS is 1.0141e-06, and RMSE is 0.0708; if VPD factors are not considered, the CC is 0.49, BIAS is 2.9364e-06, and RMSE is 0.077. Overall, the model considering VPD factors performs better in terms of correlation and error, making it more suitable for distinguishing the effects of different factors on NDVI during the study period.

### 3.3 Quantitative evaluations

Usually, we use correlation coefficient (CC), relative bias (Bias), and root mean square error (RMSE) to verify the accuracy of the model. The changing trends of NDVIc, NDVIh, and NDVIo are obtained at the pixel scale to determine the spatial distribution and impact of Cc and Ha on grasslands in the study area. Based on these trends, six response patterns of NDVI to Cc and Ha were further determined along with their contribution rates (**Supplementary Table 1**) (Zhou et al., 2022; Guo et al., 2021).

Using the partial correlation analysis method, the partial correlation coefficients between NDVIo and various meteorological factors are used to identify the impact of each Cc factor on grassland changes (**Supplementary Table 2**) (Zhou et al., 2022). The contribution rates of each driving factor are gradually stripped away in various scenarios.

## 4 Results

### 4.1 Spatiotemporal patterns of grassland changes

**Supplementary Figure 1** shows the fluctuation status of NDVI in Xinjiang from 1981 to 2015, categorizing the fluctuation status of NDVI into five levels. Among them, 8% of pixels are in a low fluctuation state, 66% of pixels are in a relatively low fluctuation state, with low fluctuation areas mainly located in the Taklimakan Desert, Kumtag Desert, and eastern Xinjiang; 4% of pixels show a higher fluctuation state (0.2> ${\text{CV}}_{\text{NDVI}}$ >=0.15), and 3% of pixels show a high fluctuation state ( ${\text{CV}}_{\text{NDVI}}$ >=0.2). Specifically, areas with higher fluctuations are mainly located in the Tianshan Mountains, Altai Mountains, and Kunlun Mountains, while other regions have lower NDVI fluctuations.

**Figure 3:**
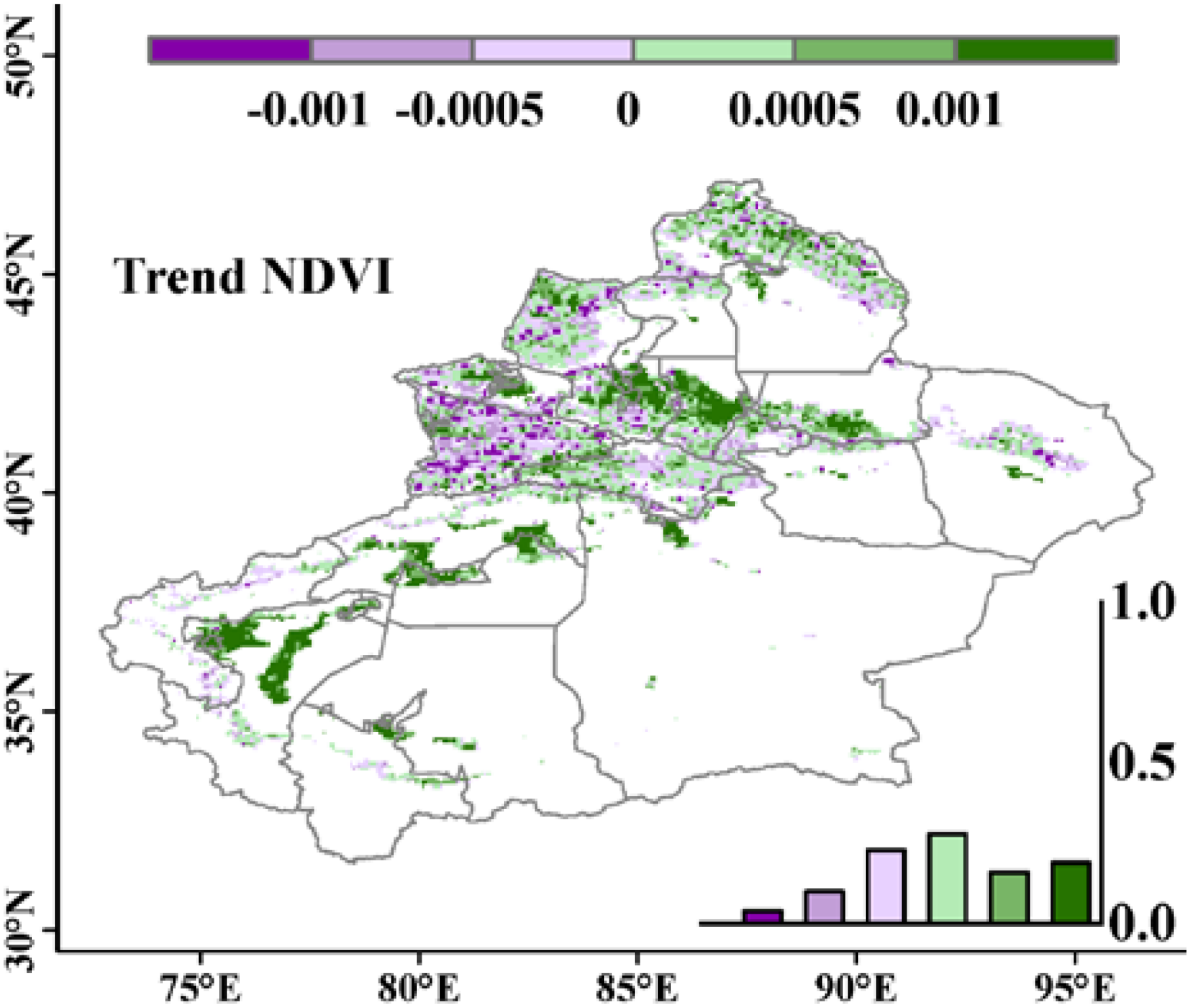
Spatial distribution of the interannual variation trend of NDVI in the study area from 1981 to 2015.The frequency histogram displaying the areal proportions (%) of corresponding coefficients is inset.

**Figure 3** shows the spatial variation trend of NDVI. From 1981 to 2015, the interannual regression coefficient distribution of NDVI in the study area ranged from -0.004 to 0.012/a. Among them, 62.5% of the regions showed an increasing trend in NDVI, indicating that NDVI gradually increased over time, while the remaining 37.5% of the regions exhibited a decreasing trend, meaning that NDVI gradually decreased over time, mainly concentrated in the western part of the study area, including the Ili River and Tekes River basins. **Supplementary Figure 2** shows that 50% of the study area’s pixel NDVI trend passed the significance test, with significant increases and decreases in NDVI distributed across the entire study area. Specifically, 36% of the regions showed a significant increasing trend in NDVI (P<0.05), with the distribution range highly overlapping with the areas where NDVI trend >0.001 in **Figure 3**; 14% of the regions exhibited a significant decreasing trend in NDVI, mainly located in the central and western parts of the study area. Generally, there was a significant spatial heterogeneity in the interannual variations of NDVI in the study area, showing an overall increasing trend.

### 4.2 Correlations between NDVI and climate variables

Using precipitation, temperature, and VPD as control variables, the correlation coefficient and partial correlation coefficient between NDVI and precipitation, temperature, and VPD were calculated. **Supplementary Figure 3** show the spatial distribution of the correlation coefficients between NDVI and precipitation, temperature, and VPD. The main relationship between NDVI and precipitation is positive (r>0), meaning that NDVI increases with increasing precipitation. The highest correlation coefficient is 0.76, the lowest is -0.62, with 29% of the study area pixels being significant. 78% of the pixels in the study area show a positive correlation, with the majority having correlation coefficients less than 0.3, mainly distributed in mountainous areas. Similarly, the dominant relationship between NDVI and temperature is positive, meaning that NDVI increases with rising temperatures. The highest correlation coefficient is 0.78, while the lowest is -0.72. 25% of the pixels in the study area show significant performance, with 69% exhibiting a positive correlation, mostly falling within the range of 0 to 0.3. 31% of the pixels show a negative correlation, indicating that NDVI decreases as temperature rises, generally exceeding -0.3, mainly concentrated in the Tacheng region. As for VPD, the number of pixels showing positive and negative correlations with NDVI is roughly equal, with the highest correlation coefficient being 0.78 and the lowest being -0.81. 30% of the pixels show significant correlation. Among these, 57% exhibit a negative correlation, where the trend of NDVI and VPD changes oppositely, primarily distributed in the western part of the study area, the central part of the Tianshan Mountains, and the southern part of the Altai Mountains. 43% of the pixels show a positive correlation, where the trend of NDVI and VPD changes in the same direction, with 64% of the pixels falling within the range of -0.3 to 0.3 overall.

**Figure 4:**
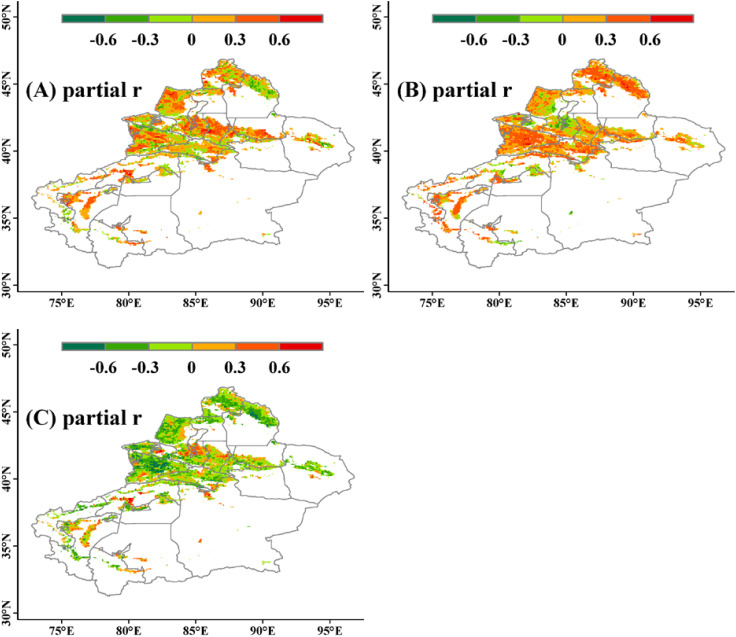
Spatial distribution of partial correlation coefficients between NDVI and climate variables. **(A–C)** represent the partial correlation coefficients between NDVI and precipitation, temperature, and VPD, respectively.

In addition, the partial correlation coefficient is used to measure the relationship between NDVI and precipitation, temperature, and VPD. **Figure 4** shows the spatial distribution of the corresponding partial correlation coefficients. The partial correlation coefficient between NDVI and precipitation ranges from -0.67 to 0.81, indicating a positive correlation in the study area, accounting for 69% of the area. The partial correlation coefficient between NDVI and temperature ranges from -0.59 to 0.82, showing a positive correlation, accounting for 83%. As for VPD, the minimum partial correlation coefficient is -0.85, and the maximum is 0.79, indicating a negative correlation between NDVI and VPD, accounting for 73% of the area. Overall, NDVI showed a stronger correlation with temperature compared to precipitation, and was negatively correlated with VPD.

### 4.3 Attributions of grassland changes

**Figure 5:**
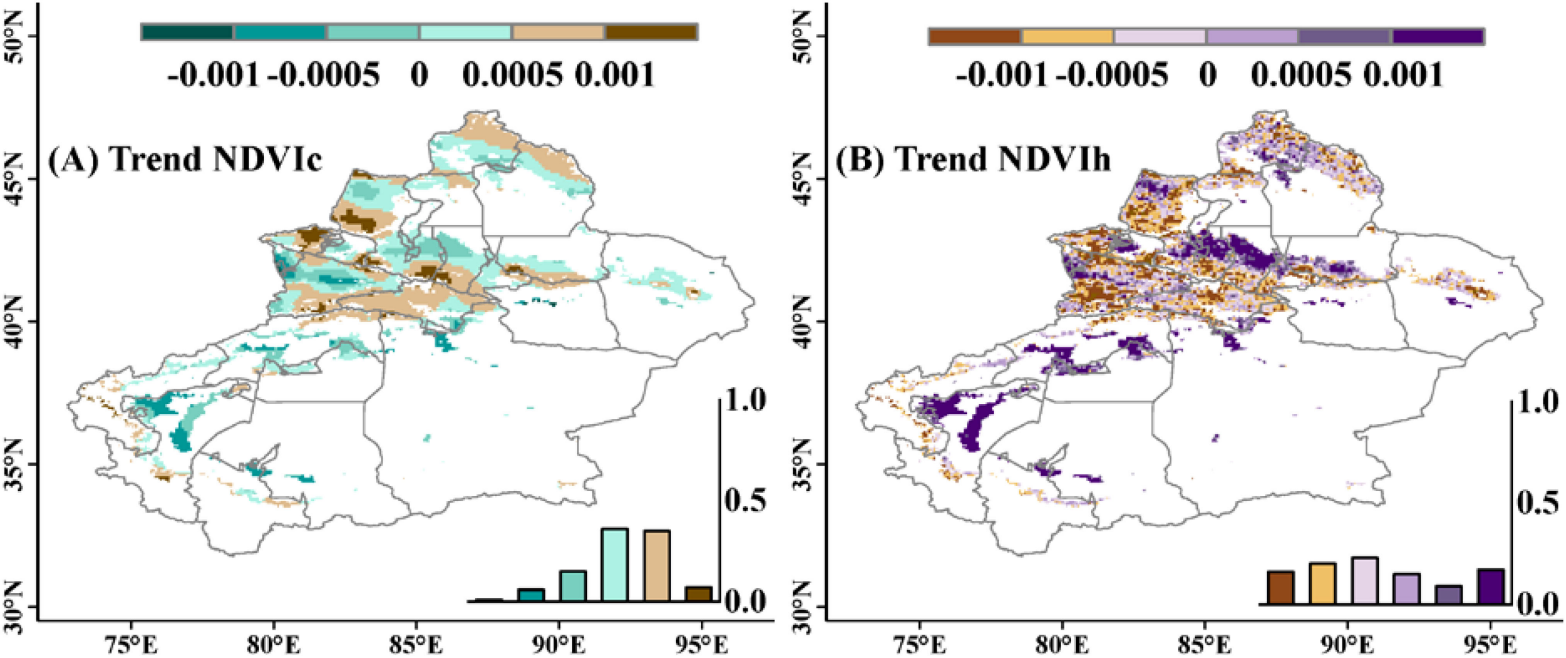
Spatial distribution of the interannual variation trends in grassland NDVI in Xinjiang from 1981 to 2015. **(A, B)** reflect the variation trends of NDVIc and NDVIh, respectively. The frequency histogram displaying the areal proportions (%) of corresponding coefficients is inset.

By establishing the grassland NDVI model, the values and spatial distribution of NDVIc and NDVIh in the study area from 1981 to 2015 were calculated. **Figure 5** shows the spatial distribution of the changing trends of NDVIc and NDVIh. NDVIc overall shows a gradually increasing trend over time, accounting for 77.7% of the study area, mainly distributed in the southern Tianshan Mountains and northern Altai Mountains; the remaining 22.3% of pixels show a decreasing trend year by year, with the declining areas evenly distributed within the study area. In contrast to NDVIc, NDVIh exhibits a widespread decreasing trend, covering 60% of the study area. The remaining 40% of pixels show an increasing trend year by year, mainly distributed in the western part of Changji Hui Autonomous Prefecture, downstream of the Yarkant River, and near Kashgar City.

**Figure 6:**
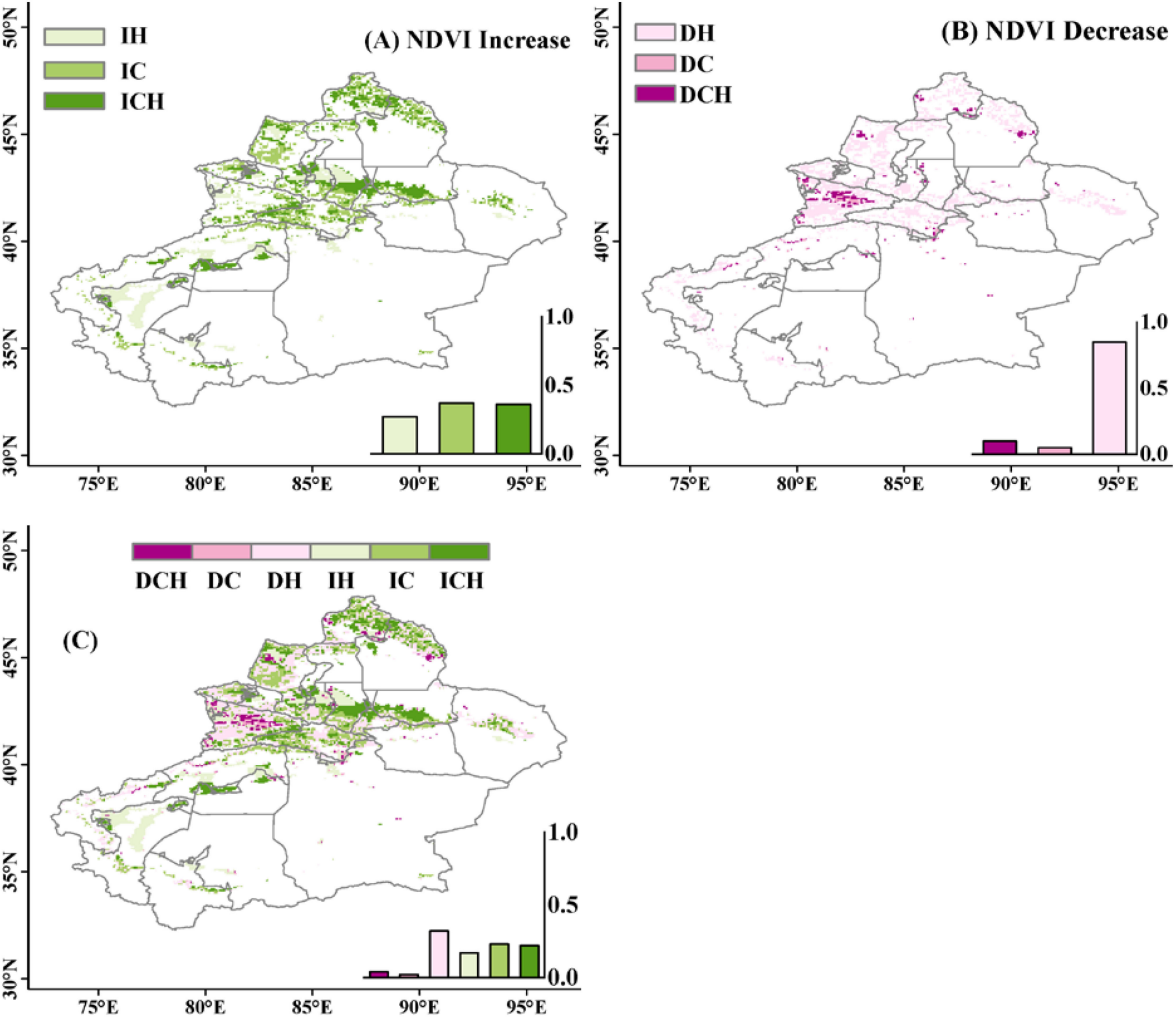
Spatial distribution of grassland NDVI response patterns. **(A–C)** reflect the overall situation of NDVI decrease, increase, and total change, respectively. In these panels, IH, IC, and ICH represent grassland increase caused by human activities, climate change, and the combined effect of climate change and human activities, respectively; DH, DC, and DCH represent grassland decrease caused by human activities, climate change, and the combined effect of climate change and human activities, respectively. The frequency histogram displaying the areal proportions (%) of corresponding regions is inset.

This study identified six response patterns of grassland NDVI to climate change and human activities through the interannual variation trends of NDVIc, NDVIo, and NDVIh. The spatial results are shown in **Figure 6**. In terms of grassland growth, the promotion effects of climate change-induced pattern (Cc), human activities-induced pattern (Ha), and climate change combined with human activities-induced pattern (Cc-Ha) on grasslands account for 37%, 28%, and 35% of the increased area, respectively. In terms of grassland reduction, it is mainly influenced by human activities (Ha), accounting for 85% of the reduced area. Other patterns Cc and Cc-Ha account for 5% and 10% of the reduced area, mainly distributed near the Tekes River and Ili River. The entire study area is dominated by an increase in grassland NDVI, accounting for 62.5% of the area. The remaining 37.5% of pixel grassland growth is inhibited, leading to a decrease in grassland NDVI, with grassland NDVI responding most significantly to pattern Ha, accounting for 32% of the study area. Overall, climate change contributed as much as human activity in terms of grassland growth, while the later one played a larger role for the grassland reduction.

### 4.4 Contributions of Cc and Ha to grassland changes

**Figure 7:**
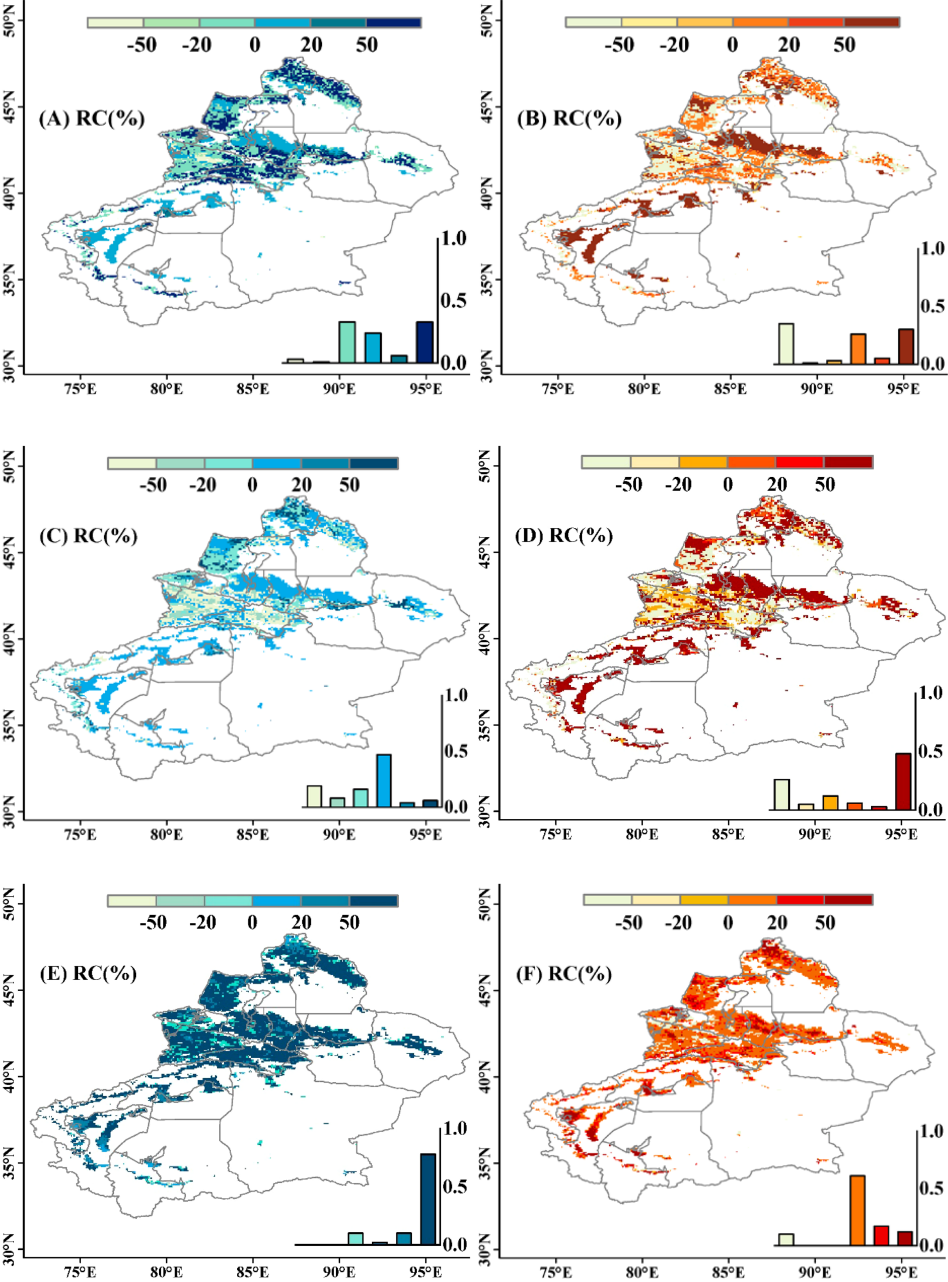
Spatial distribution of the relative contribution of climate change (left panels) and human activities (right panels) to interannual variation in grassland NDVI in Xinjiang during 1981-2015 **(A, B)**, 1981-2000 **(C, D)** and 2001-2015 **(E, F)**. A positive contribution rate indicates a trend of grassland increase, while a negative contribution rate indicates a trend of grassland decrease. The frequency histogram displaying the areal proportions (%) of corresponding coefficients is inset.

According to **Supplementary Table 2**, the contribution rates of climate change (Cc) and human activities (Ha) to grassland changes are obtained. **Figure 7** shows the spatial distribution of the contributions. The results indicate that form 1981-2015, climate change mainly affects 32% of pixels, where grassland changes show an increasing trend, meaning that the contribution rate of Cc is greater than Ha, promoting grassland growth. The main contribution area of Cc to grassland decrease is small, accounting for 3%, mainly concentrated in the Ili River Basin. For human activities, in both cases of grassland growth and decrease, the number of pixels making the main contribution is roughly the same. 29% of pixels contribute to grassland growth, with Ha’s contribution rate greater than Cc, indicating a growth trend in grassland; while pixels contributing mainly to grassland decrease account for 35%. Overall, Cc and Ha contribute 35.71% and 64.29% respectively to the total amount of grassland changes during the entire study period (**Figures 7A, B**). It should be noted that the contributions of climatic variables and human activities to grassland changes varied with time, because of enhanced climate changes and developments of the projects and policies. Hence, a temporal difference of the contributions was further explored before and after the 2000 year. The results showed that the positive contributions of climate change obviously enhanced from 80s-90s to 20s-21s (**Figure 7C, E**). The main contributing area (relative contribution > 50%) accounted for 78% of the study area after the 2000 year. Meanwhile, the negative contributions of human activities also reduced from 80s-90s to 20s-21s, especially in the areas with Ha< 50% (**Figure 7D, F**). The positive Ha is widespread across the study area after 2000, but most of them had a low contribution of between 0 and 20%.

**Figure 8:**
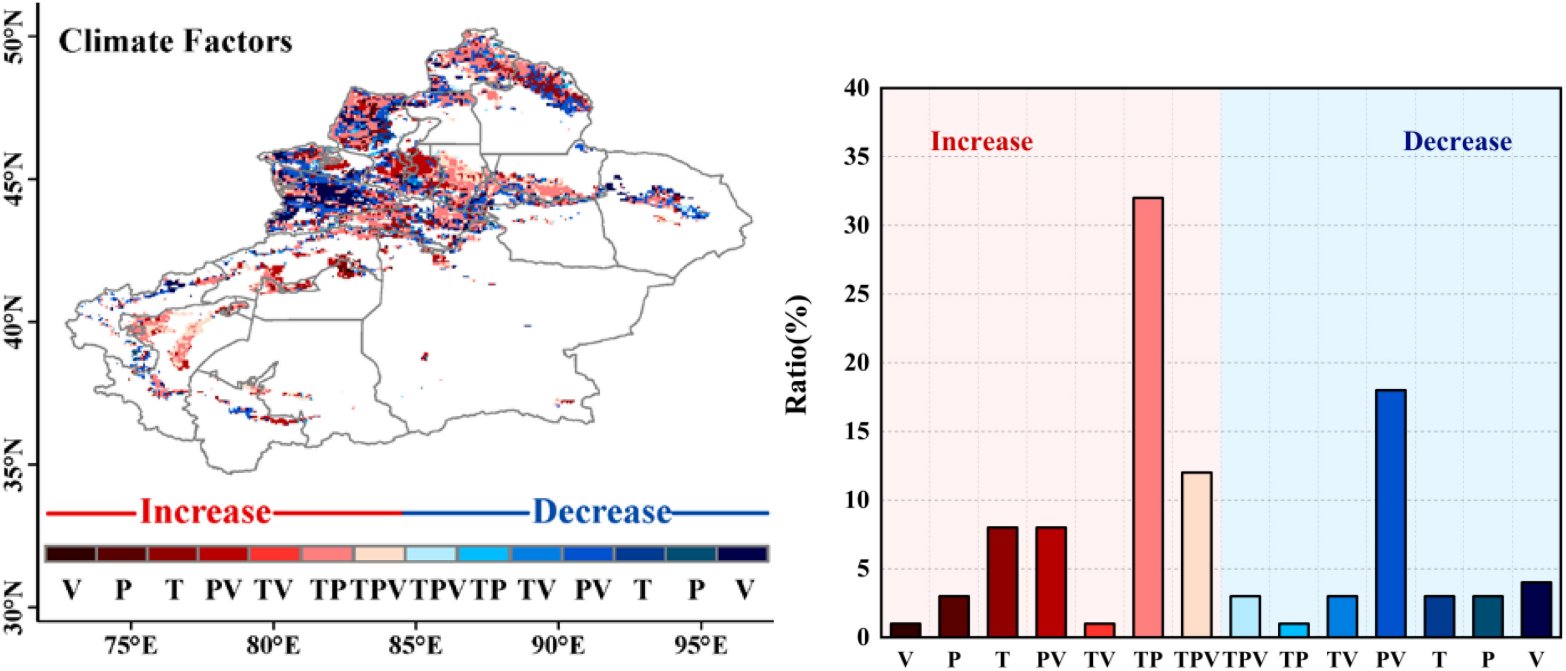
Spatial distribution of climatic drivers of grassland NDVI and frequency histogram of area proportions. The red colors indicate an increasing trend, and the blue colors indicate a decreasing trend. (P-V) represents the combined effect of precipitation and VPD, (T-V) represents temperature and VPD, (T-P) represents temperature and precipitation, and (T-P-V) represents the combined effect of temperature, precipitation, and VPD.

Based on the partial correlation coefficients of temperature, precipitation, VPD with NDVI and their identification criteria (**Supplementary Table 2**), the spatial patterns of driving factors were plotted as shown in **Figure 8**. In the case of grassland growth, the range of the combined impact of temperature and precipitation (TP) is greater than other climatic variables, accounting for 32% of the study area, mainly distributed in the Tianshan Mountains, northern part of the Kaidu River, near the Ili River, downstream of the Yarkant River, and around Kashgar City; followed by the composite pattern of temperature, precipitation, and VPD (TPV), accounting for 12%. For grasslands showing a decreasing trend, they are mainly driven by precipitation and VPD (PV), accounting for 18% of the study area, concentrated in the southern part of the Bortala Mongol Autonomous Prefecture. In general, human activities contributed more than climate change, including grazing and urbanization, in mountainous and flat areas.

## 5 Discussion

### 5.1 Spatiotemporal patterns of NDVI, NDVIc, and NDVIh

Based on the fluctuation status of NDVI, the results of this study show that the NDVI in the research area mainly remains in a low fluctuation state and a relatively low fluctuation state, indicating that from 1981 to 2015, the fluctuation degree was low, data distribution was concentrated, time series were stable, and reliability was high. This helps to improve the accuracy and effectiveness of data analysis, as well as the credibility and interpretability in practical applications. Precipitation in Xinjiang is generally low, and the arid and semi-arid climate suppresses vegetation growth, resulting in sparse vegetation cover, low and relatively stable NDVI values (Liu et al., 2016). The fluctuation status in the Tianshan Mountains and Altai Mountains is higher due to sufficient moisture and higher temperatures in these areas, which extend the vegetation growth period, leading to more luxuriant vegetation during the growing season, with greater fluctuations between the maximum and minimum NDVI values (Liu et al., 2016).

50% of the research area pixel NDVI change trend was tested for significance, indicating a large magnitude of data change and strong reliability. The entire research area exhibits spatial heterogeneity in NDVI, showing an overall upward trend. The areas with increasing NDVI mainly include the western part of Changji Hui Autonomous Prefecture, the southern part of Tacheng Prefecture, and the northwestern part of the Tarim Basin, accounting for 62.5% of the research area. The decreasing areas are mainly distributed in the western part of the research area, as well as the Ili River and Tekes River basins, accounting for 37.5% of the research area. This is mainly attributed to the warming and humidifying trend of the climate in Xinjiang in recent years, where increased precipitation and rising temperatures have promoted grassland growth (Li et al., 2018; Zhang et al., 2021; Yao et al., 2018). Research by Zhang et al. (2018) on the dynamic response of grassland to climate change and human activities in Xinjiang shows that the grasslands in the Ili River Valley are experiencing a degradation trend, similar to the results of this study (Zhang et al., 2018). Furthermore, it indicates that the degraded grassland area is gradually spreading towards the western part of the Tianshan Mountains in terms of spatial distribution. Additionally, the turning-point has been detected for the NDVI series over the study area. It shows that the increasing trend of NDVI changed after the year of 2000. However, there was a little difference between the slopes before and after the turning point, and thereby it was not considered in the present work.

Through the grassland NDVI model, the spatiotemporal patterns of NDVIc (NDVI affected by climate change) and NDVIh (NDVI affected by human activities) in the study area were analyzed. The results show that NDVIc generally increases over time, indicating a growing role of climate change in promoting grassland growth year by year, accounting for 77.7% of the study area, with 41.7% of pixels greater than 0.0005/a. The growth areas mainly include the Tianshan Mountains, Altai Mountains, Irtysh River Basin, Tacheng area, and Bortala Mongol Autonomous Prefecture. This may be related to climate change and topographical fluctuations in Xinjiang: on the one hand, there has been a trend of warming and increased humidity in Xinjiang in recent years (Wang et al., 2020), with increased precipitation and thereby soil moisture helping meet the water needs of grassland growth, rising temperatures promoting grassland photosynthesis, improving photosynthetic efficiency, and thus enhancing grassland growth; on the other hand, the closer to mountainous areas or the larger the mountains, the greater the trend of increasing annual precipitation, as moist air forced to rise when passing through mountains leads to cooling with altitude, making water vapor in the air more likely to condense into precipitation, promoting grassland around mountainous and basin areas, thereby improving degradation (Yao et al., 2022).

In contrast to NDVIc, NDVIh mainly shows a decreasing trend, indicating that human activities are increasingly inhibiting grassland growth year by year, accounting for 60% of the study area. About 37% of the pixels have a decrease rate of less than -0.0005/a, which is lower than the increase rate of NDVIc. The remaining 40% of pixels show an increasing trend. The areas where NDVIh is increasing year by year are mainly distributed in the western part of Changji Hui Autonomous Prefecture, downstream of Yarkant River, Aksu City, Xinhe City, Korla City, and near Kashgar City. This is related to land use changes, conservation and restoration projects, and water resource management: human activities have led to changes in land use, such as an increase in parks, green spaces, or protected areas; at the same time, irrigation systems or other water projects have a restorative effect on the ecological environment, promoting grassland growth.

### 5.2 Effects of climatic variables to grass growth

In order to eliminate the influence of other variables, partial correlation coefficients were used to measure the relationship between NDVI and precipitation, temperature, and VPD. The results show that compared to the correlation coefficient, in the study area, NDVI is mainly positively correlated with precipitation. That is, in areas with higher precipitation, the NDVI values are also higher, accounting for a relatively low proportion of 69%, with a distribution range roughly the same, mainly distributed in mountainous areas. Moreover, the number of pixels showing a negative correlation in the southern part of the mountains increases, indicating that moisture conditions are an important factor affecting the variation of grassland NDVI, as soil moisture is one of the essential resources for plant photosynthesis and growth, and an increase in soil moisture leads to an increase in NDVI values (Liang et al., 2015). It should be noted that NDVI is calculated from the reflectance values of the near-infrared and red visible light bands, with the near-infrared band being a strong absorption region for water bodies. When precipitation causes significant changes in soil moisture content, the NDVI values decrease. Therefore, in some areas, there is a negative correlation between NDVI and precipitation (Li et al., 2011).

NDVI is mainly positively correlated with temperature, accounting for 83%, slightly higher than the correlation coefficient of 69%, and the correlation strength is stronger than that with precipitation. The number of negatively correlated pixels in the western part of Tacheng region is smaller, mainly located in its southern part. Specifically, it reflects the sensitivity of vegetation activity in mid-high latitude zones to heat factors: on the one hand, most areas in the study area belong to arid or semi-arid climates, with relatively low precipitation. Under such climatic conditions, the impact of temperature on vegetation growth is more significant than that of precipitation, promoting organic matter decomposition and improving the effectiveness of vegetation nutrient utilization; on the other hand, the increase in temperature leads to the evaporation of soil moisture in water-rich areas, maintaining high air humidity, improving local microclimates, and increasing NDVI (Huang et al., 2022).

For VPD, unlike the correlation coefficient, it is mainly negatively correlated with NDVI. That is, NDVI increases as VPD decreases and decreases as VPD increases, accounting for 73%, much higher than the correlation coefficient of 57%. Moreover, the number of pixels showing a negative correlation in the southern Altai Mountains and the Tianshan Mountains has significantly increased. The reason is that when VPD is low, the air is humid, which can suppress transpiration and surface evaporation, beneficial for soil moisture retention and organic matter accumulation (Huang et al., 2022), thereby promoting grassland growth. In regions where NDVI is positively correlated with VPD, high VPD is usually associated with clear and dry weather conditions, meaning sufficient sunlight and suitable temperatures. Compared to the inhibitory effect of VPD, these weather conditions have a stronger promoting effect on plant growth.

In summary, the changes in precipitation, temperature, and VPD are closely related to the variation of NDVI in the study area. Considering the individual impacts of precipitation, temperature, and VPD, NDVI is mainly positively correlated with precipitation and temperature, and negatively correlated with VPD. Among them, the region where NDVI is positively correlated with temperature has a larger area and a stronger relationship, indicating that temperature has a greater impact on the vegetation coverage of grasslands in the study area.

For the same pixel, the partial correlation coefficient and the correlation coefficient show different relationships, indicating that there are complex composite relationships between NDVI and precipitation, temperature, and VPD. After controlling for other variables, the direct relationship between NDVI and the remaining variables may exhibit an opposite trend. To further explore the impact of climate variables on grassland NDVI changes, identify the individual and composite contributions of climate variables, and generate spatial pattern maps of driving factors. The results indicate that grasslands in the study area are more influenced by the combined effects of multiple climate variables than by the effects of a single climate variable. Combining the contribution rates of climate variables to grassland changes (**Figure 7**), it is mainly found that Cc promotes grassland recovery. In terms of individual climate variables, the influencing factors from largest to smallest are temperature, VPD, and precipitation, meaning that temperature has a greater impact on grassland changes, which is consistent with the above conclusion.

In regions where there is a growing trend in grassland, the main factor is the combined influence of temperature and precipitation (TP). That is, the composite pattern of temperature and precipitation is the main factor for grassland improvement, as grass growth depends on both water and temperature, and neither can be lacking.

In areas showing a decreasing trend in grassland, the main influence is from the combined effects of precipitation and VPD (PV), meaning that the composite pattern of precipitation and VPD has the most significant driving effect on grassland degradation. As most parts of Xinjiang are in arid or semi-arid climates with low annual precipitation, high VPD indicates strong air absorption capacity for moisture, leading to rapid evaporation of soil moisture and exacerbating water stress in grasslands.

### 5.3 Relative contributions to grassland changes

Based on the spatial distribution map of six response modes to climate change and human activities based on grassland NDVI, it can be seen that for grassland growth, it is influenced by both climate change and human activities, with a relatively equal degree of impact. The contributions of climate change and human activities are 52% and 48%, respectively. Regions with higher contributions from climate change are mainly distributed in the Altai Mountains, western Tacheng area, Kaidu River Basin, as well as the junction of Turpan City and Changji Hui Autonomous Prefecture; regions with higher contributions from human activities are located in the northern part of the Tianshan Mountains, western Tarim Basin, and the northern part. Grassland growth on the edge of the Tarim Basin and the southern edge of the Junggar Basin mainly benefits from human activities. The sand control and desertification control projects implemented after the National Sand Control Conference in 1991, including the construction of a sand control system with shrubs and grasses and measures such as fencing and enclosure of desert grasslands, have improved soil quality, provided suitable growth environments, and promoted grassland growth. The ecological governance project around the Tarim Basin launched in 2009 and the sand control and desertification control project in the Junggar Basin further accelerated the recovery of grasslands. In addition, the settlement project for herdsmen implemented in 1986, the national grazing ban and grassland restoration project in 2002, the grassland ecological compensation incentive mechanism in 2011, all played a role in promoting grassland growth (Cai et al., 2022).

Human activities (Ha) are the main driving factor for the reduction of grasslands, such as urban expansion, mining, grazing, and agricultural activities, which inhibit grass growth, leading to a decrease in biomass, and they also occupy the largest area of the entire study area (32%). In this study, the main cause of grassland degradation and desertification is grazing (Yang et al., 2014). The Altai Mountains, Tacheng region, and Ili River Valley are regions with high grazing rates in Xinjiang (Huang et al., 2018), so they are also concentrated areas where human activities lead to a decrease in NDVI. Notably, our present approach could technologically distinguish the contributions of the climatic variables and non-climatic variables to the variations of NDVI over the grasslands. However, it is difficult to identify what kind of human activities dominates contribution without corresponding driving data. Hence, we tried to investigate the relationship between the contribution of human activities and the grazing intensity obtained from a recently released data (Wang et al., 2024). We found that the negative contributions were quite consistent with the increasing grazing intensity across the study area. In contrast, the positive contributions were mainly associated to the reducing grazing. This generally support our results and infer.

Based on the results of the relative contributions of climate change and human activities to grassland NDVI changes, it is shown that human activities contribute more (64.29%) to grassland changes than climate change (35.71%). These results are generally consistent with the previous studies which investigated grassland dynamics in response to climate change and human activities in Xinjiang or the (semi-) arid areas of China (Zhang et al., 2018; Yang et al., 2017; Xue et al., 2023). This is because the study area is mainly composed of mountains, river basins, and oases. For mountainous areas, due to the complex and varied terrain in mountainous regions, as well as significant differences in climate and human environment between the southern and northern slopes, grasslands are more driven by non-climatic factors than climatic factors (Zhang et al., 2018). In river basins and oasis areas, due to the fact that the river valleys and areas around oases are the main grazing and pasture areas, human activities are significant, so human activities contribute more to grassland changes (Huang et al., 2022).

### 5.4 Uncertainties and further studies

Although the aims were generally achieved, there remain some limitations in our work. First, the GIMMS dataset offers a spatially continuous and long temporal span NDVI data, its spatial resolution is relatively coarse, which may impact the detections of vegetation growth to some extent. Remotely sensed data with a higher spatial resolution would be needed for a finer analysis. Second, the relative contributions of climate change and human activities were quantified, while the approach adopted in this study is still an empirical-based statistical analysis. Finally, it could not identify what kind of human activities dominates the contribution. A robust casual analysis or model simulation may be helpful to crack these nuts.

## 6 Conclusions

This article systematically studied the spatiotemporal patterns and fluctuation status of grasslands in Xinjiang from 1981 to 2015, revealing the correlation between NDVI and precipitation, temperature, and VPD. By combining six response modes of NDVI changes, it quantitatively analyzed the contributions of climate change and human activities to grassland changes. The study found that the NDVI of Xinjiang grasslands exhibits spatial heterogeneity, with an overall upward trend. This is mainly attributed to the warming and humidifying trend of Xinjiang’s climate in recent years, where increased precipitation and higher temperatures have promoted grass growth. The regions with increasing NDVI are mainly concentrated in the western part of Changji Hui Autonomous Prefecture, the southern part of Tacheng area, and the northwestern part of the Tarim Basin; while the decreasing areas are mainly distributed in the western part of the study area, the Ili River, and the Tekes River basin. Further analysis shows that temperature and precipitation (TP) have the most significant impact on grassland growth, while precipitation and VPD (PV) have the greatest impact on grassland reduction. For grassland growth, the contributions of climate change and human activities are roughly equivalent; however, for grassland reduction, the influence of human activities is more pronounced. Overall, in mountainous and flat areas, human activities have a greater contribution to grassland changes than climate change. Therefore, when predicting future trends in grassland ecosystem changes, it is necessary to consider the quantitative impacts of both climate change and human activities simultaneously.

## Data Availability

The original contributions presented in the study are included in the article/**Supplementary Material**. Further inquiries can be directed to the corresponding author.
