# Supplementary material for: Quantitative impacts of climate change and human activities on grassland growth in Xinjiang, China
Source: Front Plant Sci. 2025 Jan 22;15:1497248. doi: 10.3389/fpls.2024.1497248 (PMC11795316; doi:10.3389/fpls.2024.1497248)
Supplement: Supplementary file 1 [file SupplementaryFile1.docx]

Supplementary Material

# Supplementary Figures and Tables

## Supplementary Figures


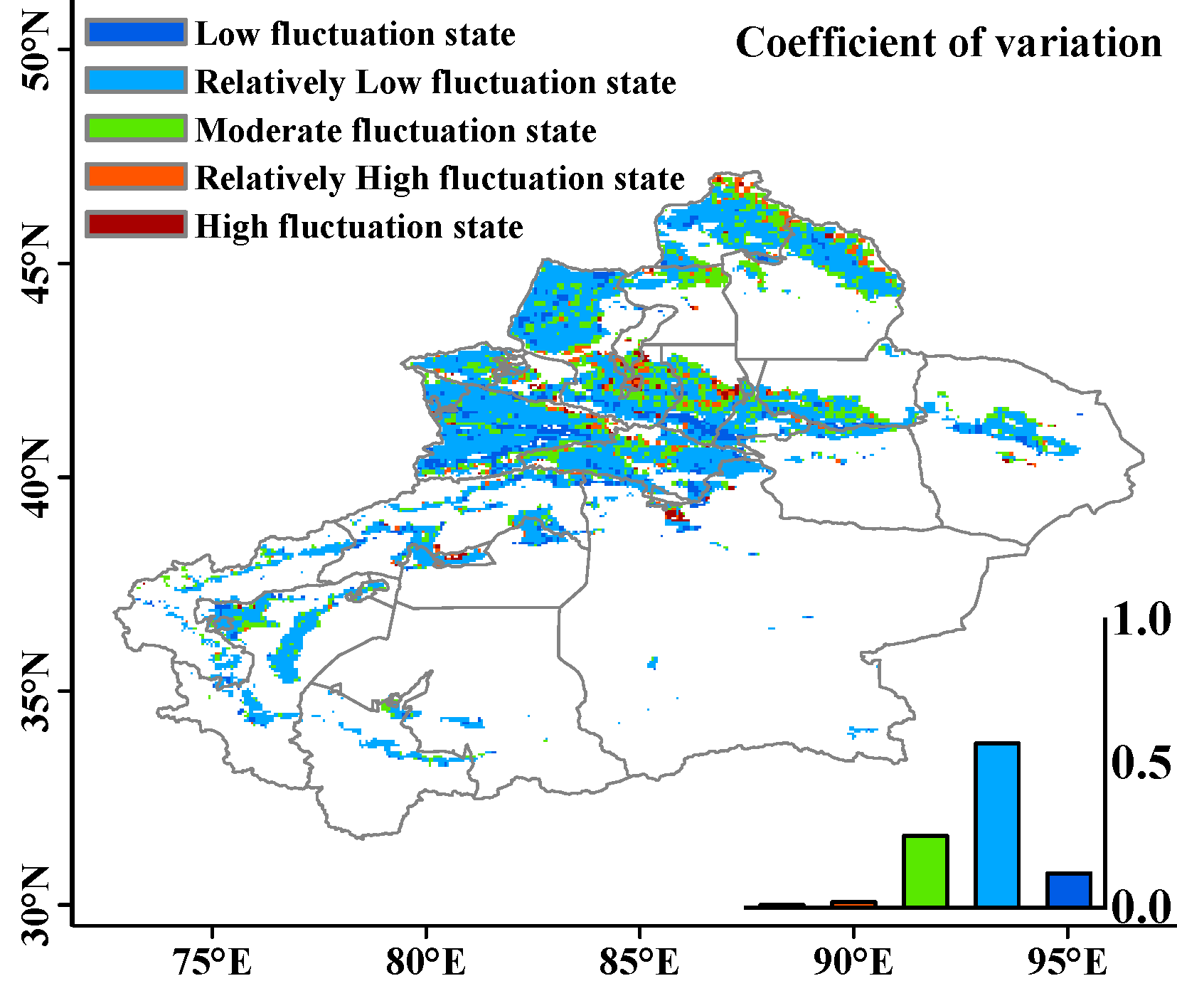


**Supplementary Figure 1.** Spatial distribution of NDVI fluctuation state in the study area from 1981 to 2015.The frequency histogram displaying the areal proportions (%) of corresponding states is inset.


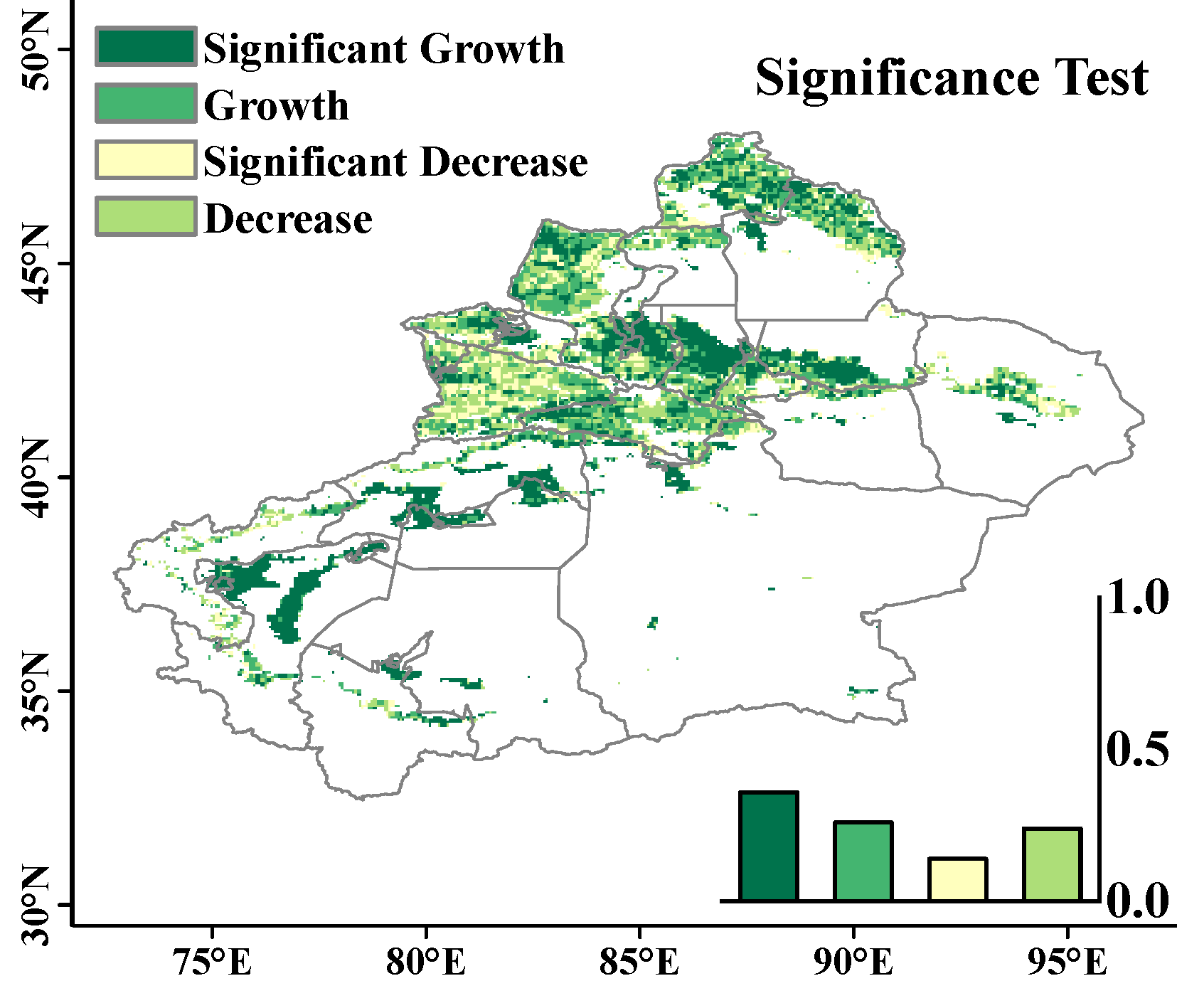


**Supplementary Figure 2.** Spatial distribution of the significance of interannual variation trends in NDVI in the study area from 1981 to 2015. Dark green and light yellow pixels indicate areas where the trends were significant at p < 0.05.The frequency histogram displaying the areal proportions (%) of corresponding regions is inset.


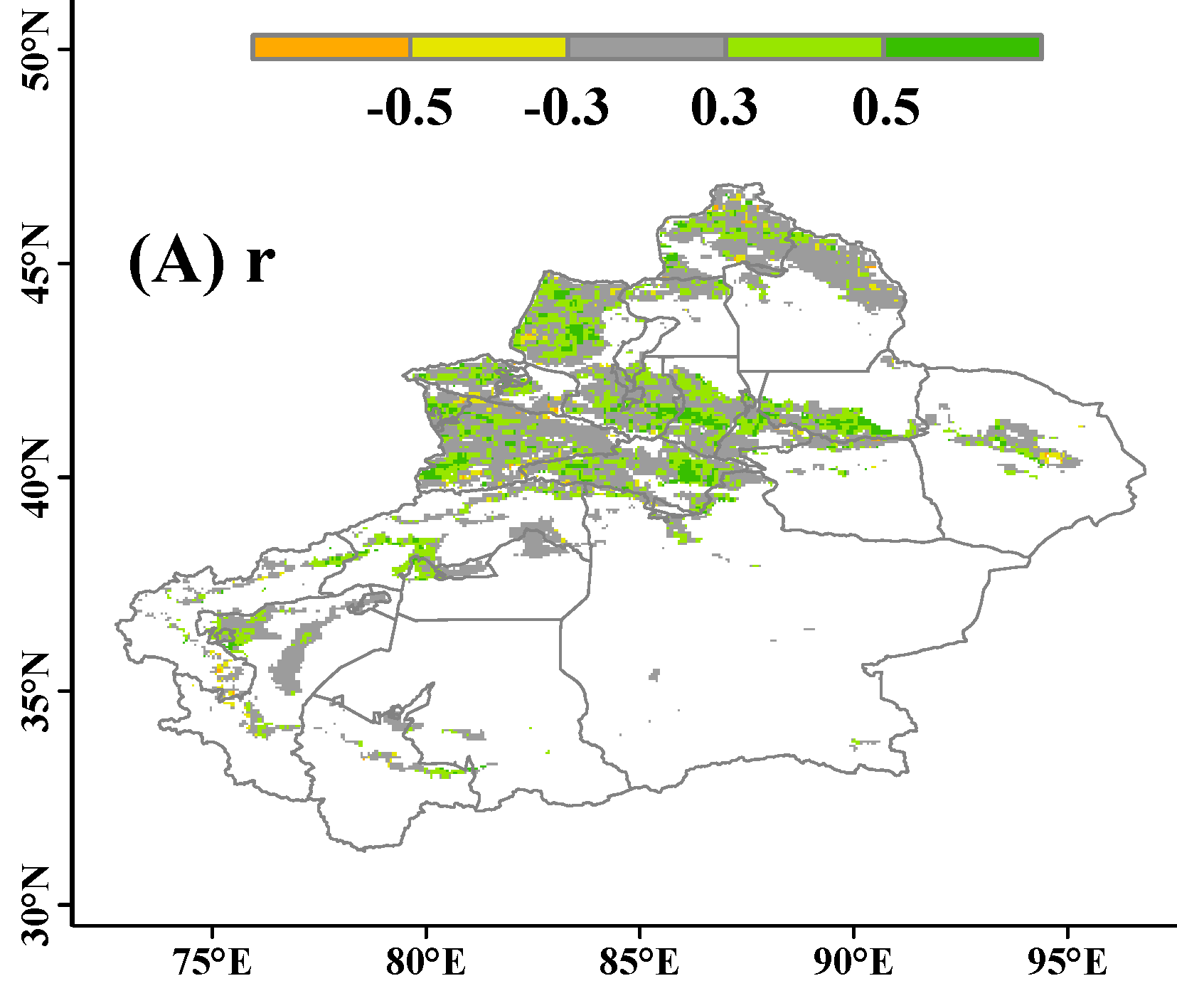

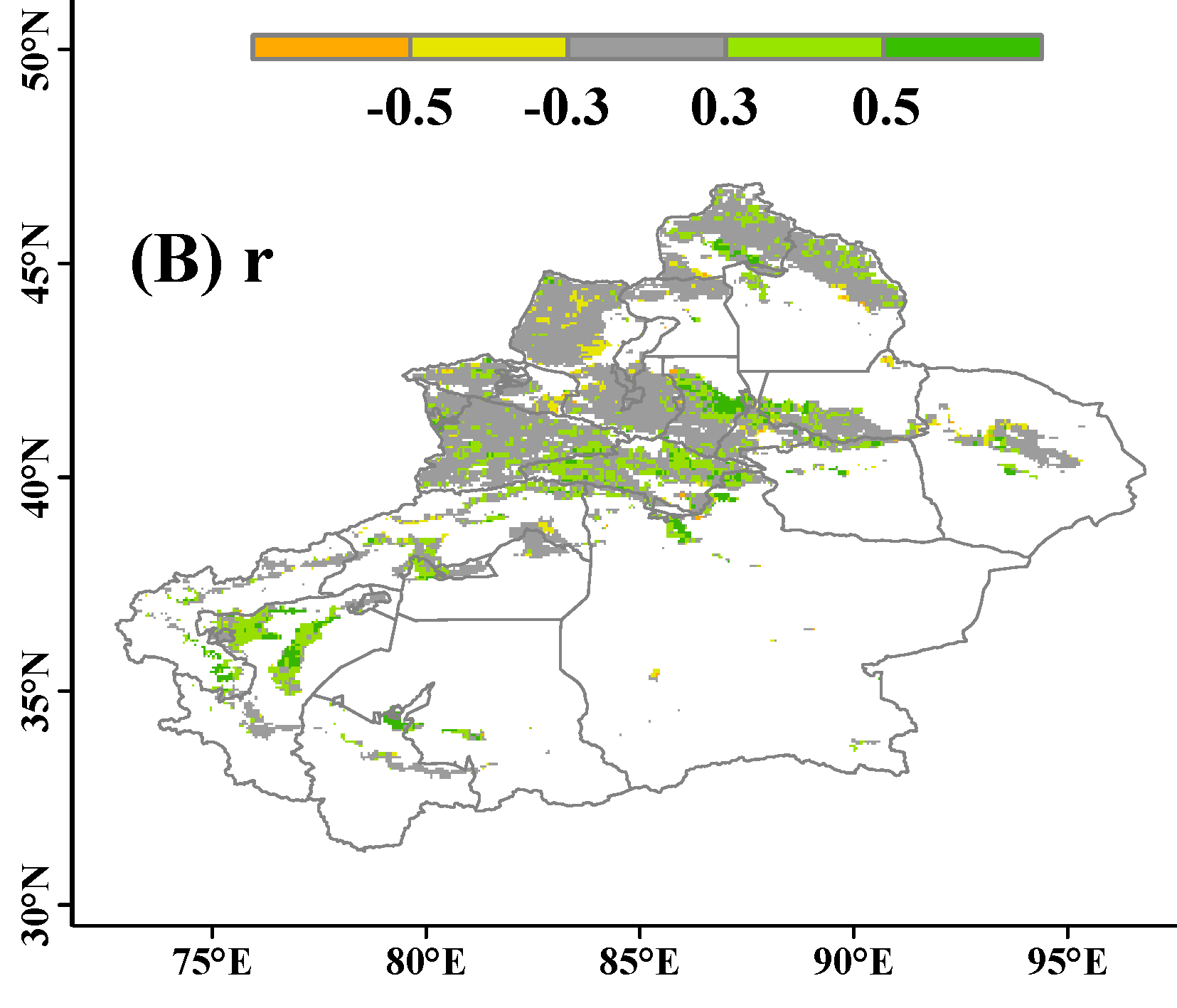

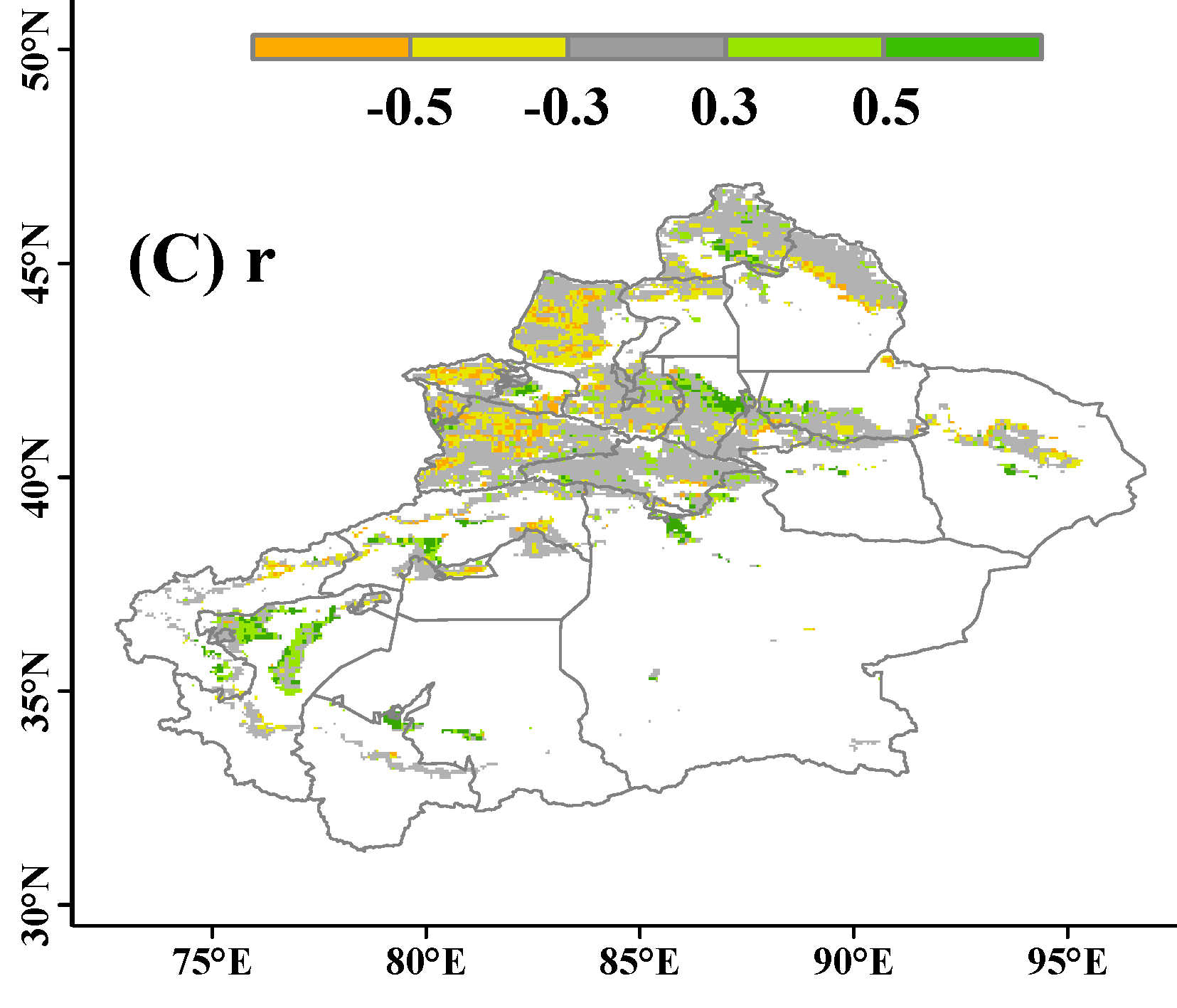


**Supplementary Figure 3.** Spatial distribution of correlation coefficients between NDVI and climate variables. Panels A, B, and C represent the correlation coefficients between NDVI and precipitation, temperature, and VPD, respectively.

## Supplementary Tables

**Supplementary Table 1.** Criteria for NDVI response pattern identification and contribution calculation. S_O_, S_C_ and S_H_ represent the slopes of observation NDVI, climate-induced NDVI and human-induced NDVI, respectively.

| $\mathrm{NDVI}_{o}$ | Vegetation trends | Division Standard | | Drivers | Contribution Rate | |
| --- | --- | --- | --- | --- | --- | --- |
|  |  | $\mathrm{NDVI}_{c}$  slope | $\mathrm{NDVI}_{h}$  slope |  | Climate | Human |
| $+$ | Increase | >0 | >0 | Both climate change  and human activities (Cc-Ha) | $\frac{S_{C}}{S_{O}}$ | $\frac{S_{H}}{S_{O}}$ |
|  |  | >0 | <0 | Climate change (Cc) | 100 | 0 |
|  |  | <0 | >0 | Human activities (Ha) | 0 | 100 |
| $-$ | Decrease | <0 | <0 | Both climate change  and human activities (Cc-Ha) | $\frac{S_{C}}{S_{O}}$ | $\frac{S_{H}}{S_{O}}$ |
|  |  | <0 | >0 | Climate change (Cc) | 100 | 0 |
|  |  | >0 | <0 | Human activities (Ha) | 0 | 100 |

**Supplementary Table 2.** Criteria for Identifying NDVI Response Patterns to Meteorological Factors.

| NDVI slope | Vegetation trend | $T_{\mathrm{PCC}}$ | $P_{\mathrm{PCC}}$ | $V_{\mathrm{PCC}}$ | Drivers |
| --- | --- | --- | --- | --- | --- |
| $+$ | Increase | $+$ | $+$ | $+$ | Together with temperature, precipitation, and VPD (T-P-V) |
|  |  | $+$ | $+$ | $-$ | Both temperature and precipitation (T-P) |
|  |  | $+$ | $-$ | $+$ | Both temperature and VPD (T-V) |
|  |  | $-$ | $+$ | $+$ | Both precipitation and VPD (P-V) |
|  |  | $+$ | $-$ | $-$ | Temperature (T) |
|  |  | $-$ | $+$ | $-$ | Precipitation (P) |
|  |  | $-$ | $-$ | $+$ | VPD(V) |
| $-$ | Decrease | $-$ | $-$ | $-$ | Together with temperature, precipitation, and VPD (T-P-V) |
|  |  | $-$ | $-$ | $+$ | Both temperature and precipitation (T-P) |
|  |  | $-$ | $+$ | $-$ | Both temperature and VPD (T-V) |
|  |  | $+$ | $-$ | $-$ | Both precipitation and VPD (P-V) |
|  |  | $-$ | $+$ | $+$ | Temperature (T) |
|  |  | $+$ | $-$ | $+$ | Precipitation (P) |
|  |  | $+$ | $+$ | $-$ | VPD(V) |
